# Dioecious hemp (*Cannabis sativa* L.) plants do not express significant sexually dimorphic morphology in the seedling stage

**DOI:** 10.1038/s41598-021-96311-w

**Published:** 2021-08-19

**Authors:** Lesley G. Campbell, Kristen Peach, Sydney B. Wizenberg

**Affiliations:** 1 Department of Chemistry and Biology, Ryerson University, 350 Victoria St, Toronto, ON M5B 2K3 Canada; 2 National Center for Ecological Analysis and Synthesis, University of California, 735 State St #300, Santa Barbara, CA USA

**Keywords:** Genetics, Plant sciences, Population genetics, Sexual selection

## Abstract

Some economically important crop species are dioecious, producing pollen and ovules on distinct, unisexual, individuals. On-the-spot diagnosis of sex is important to breeders and farmers for crop improvement and maximizing yield, yet diagnostic tools at the seedling stage are understudied and lack a scientific basis. Understanding sexual dimorphism in juvenile plants may provide key ecological, evolutionary and economic insights into dioecious plant species in addition to improving the process of crop cultivation. To address this gap in the literature, we asked: can we reliably differentiate males, females, and co-sexual individuals based on seedling morphology in *Cannabis sativa,* and do the traits used to distinguish sex at this stage vary between genotypes? To answer these questions, we collected data on phenotypic traits of 112 *C. sativa* plants (50 female, 52 male, 10 co-sexuals) from two hemp cultivars (CFX-1, CFX-2) during the second week of vegetative growth and used ANOVAs to compare morphology among sexes. We found males grew significantly longer hypocotyls than females by week 2, but this difference depended on the cultivar investigated. Preliminary evidence suggests that co-sexual plants may be distinguished from male and female plants using short hypocotyl length and seedling height, although this relationship requires more study since sample sizes of co-sexual plants were small. In one of the cultivars, two-week old male plants tend to produce longer hypocotyls than other plants, which may help to identify these plants prior to anthesis. We call for increased research effort on co-sexual plants, given their heavy economic cost in industrial contexts and rare mention in the literature. Our preliminary data suggests that short hypocotyl length may be an indicator of co-sexuality. These results are the first steps towards developing diagnostic tools for predicting sex using vegetative morphology in dioecious species and understanding how sexual dimorphism influences phenotype preceding sexual maturity.

## Introduction

Dioecious taxa represent 5–6% of all angiosperms and are found in approximately 43% of plant families^1^. Distinct from dioecious animal systems, where the germ line differentiates in embryos, plants have no differentiated germ line^2^. Instead, totipotent meristematic cells proceed through vegetative development before eventually forming differentiated flowers^3^. Sexual dimorphism is charismatic of dioecious animal species and it is generally easy to predict the sex of animal embryos once diagnostic gendered features appear in juveniles^4,5^. Contrastingly, assigning sexual identity to a plant prior to sexual maturity is difficult because diagnostic, gendered morphological features are not well described in juvenile plants. Some dioecious species are economically important, including, but not limited to: asparagus (*Asparagus officinalis* L.), hemp (*Cannabis sativa* L.), mulberry (*Morus* spp.), nutmeg (*Myristica fragrans* Houtt), date palm (*Phoenix dactylifera* L.), rattan (*Calamus* spp.), betel (*Piper betle* L.), pistachio (*Pistacia vera* L.), kiwifruit (*Actinidia deliciosa*), and yam (*Dioscorea* spp.)^6^. Some of these dioecious plants have evolved sex chromosomes, as is the case with *C. sativa*^7–9^, a species which also displays lability in sexual expression, wherein unisexual plants can develop co-sexual sex expression in response to environmental or chemical triggers^10,11^.

Early diagnosis of sex is very important to both breeders and farmers for crop improvement or production purposes, yet there is little published diagnostic morphological data to support this emerging agricultural industry (but see^12,13^). For instance, in *C. sativa*, if pollen is released near unpollinated female plants, the crop of exclusively female plants will be pollinated and is considered by federal regulators in Canada as “contaminated”^14^. In Canada pollinated cannabis can only be used for oil extract, not raw floral material, limiting market capacity. Moreover, pollinated cannabis has lower cannabinoid content, making it a less efficient way to produce oil extracts. Early morphological differences may reflect divergent life history strategies and underlying differences in the genetic architecture among male and female plants. Thus, understanding sexual dimorphism in juvenile plants may provide key economic, ecological, and evolutionary insights into dioecious plant species. Here, to complement previous reviews on the fundamental differences among dioecious plants^15^⁠, we review specific examples of sexual dimorphism in vegetative stage plants, explore whether this data fits with eco-evolutionary theory for the expression of sexual dimorphism in plants, and test whether this hypothesis allows us to predict plant sex within the first two weeks of seedling growth in an economically important species, *C. sativa*.

Sex determination is accomplished in dioecious plants via three broad mechanistic categories: genetic control^16,17^, epigenetic control^18–20^, and hormonal control^19,21^. Accordingly, many researchers have developed genetic tools to pre-emptively diagnose plant sex^22–25^, and scientists regularly develop molecular markers to assign gender to seedlings of widely cultivated, dioecious crops prior to flowering^25–27^. However, there are two problems with relying on molecular markers for predicting sex. First, the technology to perform molecular studies in situ is still expensive enough that diagnostic tools are unaffordable for most growers and breeders. Second, despite sex expression often being driven by genetic or epigenetic expression, labile sex expression is predicted to be adaptive in variable environments^28,29^⁠. Thus, morphological markers in seedlings of future sex expression may be more accurate predictors of plant sex, given the environmental context in which plants are grown, and more affordable.

Sexual dimorphism in dioecious species is most frequently described in adult, flowering plants because of the obvious floral differences^30–32^, and because the morphology of young plants is unmeasured and thus undocumented^33,34^. This may be because sexual dimorphism is not expressed in seeds, seedlings, or vegetative plants, or is not sufficiently described to allow the use of morphological sex markers^15^. Rare comparisons of plants in experimental contexts suggest there may be differences in male and female seedlings, including competitive differences^35^ and differences in their gene expression^11,36^. Genetic linkage, epigenetic linkage, or phenotypic consequences of plant hormone release may cause correlated sex and vegetative trait expression^37–39^. Alternatively, juvenile sexual dimorphism could be a consequence of adaptive differences in resource allocation^40–42^. Sexual dimorphism brought upon by differential costs of reproduction can induce early resource partitioning in response to somatic investment in reproductive success^42^, influencing expression of vegetative traits (i.e. seedling morphology), but would be more likely to occur in annual species such as *C. sativa*^6^, who require earlier investment due to their shortened life cycle. These adaptive differences may be interpreted using Rensch’s rule, wherein sexual dimorphism increases with body size when males are the larger sex and decreases when females are^35,43^.

Sexually dimorphic traits in seed or seedling stages are occasionally reported^44–46^. In addition to demographic differences^44,45^, sex-based morphological differences can manifest in more obvious ways, such as some physiological processes (the production and accumulation of phytohormones), growth, relative size, and morphology of leaf and stem characteristics^47,48^. *Spinacia oleracea*, a dioecious annual, displays sexual dimorphism during reproductive maturity^46^ but morphological differences during other stages have also been observed. Size dimorphism in the seeds of *S. oleraceae*^49^ could be evident of adaptive differences in resource allocation by mothers that recognize differential costs of reproductive success between male and female seedlings^40,42^. In one well studied dioecious plant, *Silene latifolia*, with strongly dimorphic flowers^50^⁠, plants are approximately similar in size during vegetative growth and it is only with measuring gene expression that one can distinguish genders prior to flowering^51^⁠. Finally, height dimorphism of *Rumex hastatulus*, changed predictably during the life cycle of this wind pollinated plant, with males taller than females at flowering and the reverse pattern occurring during seed maturation^52^⁠. Thus, we predict sex-linked differences in seedling height and leaf morphology of juvenile *C. sativa* plants coinciding with the observed dimorphism documented in other species. As a result, we ask: can we reliably differentiate males, females, and co-sexuals based on seedling morphology in *C. sativa*? And do the traits used to distinguish sex at this stage vary between cultivars?

## Results

### Distinguishing among male and female *Cannabis* seedlings

**Figure 1:**
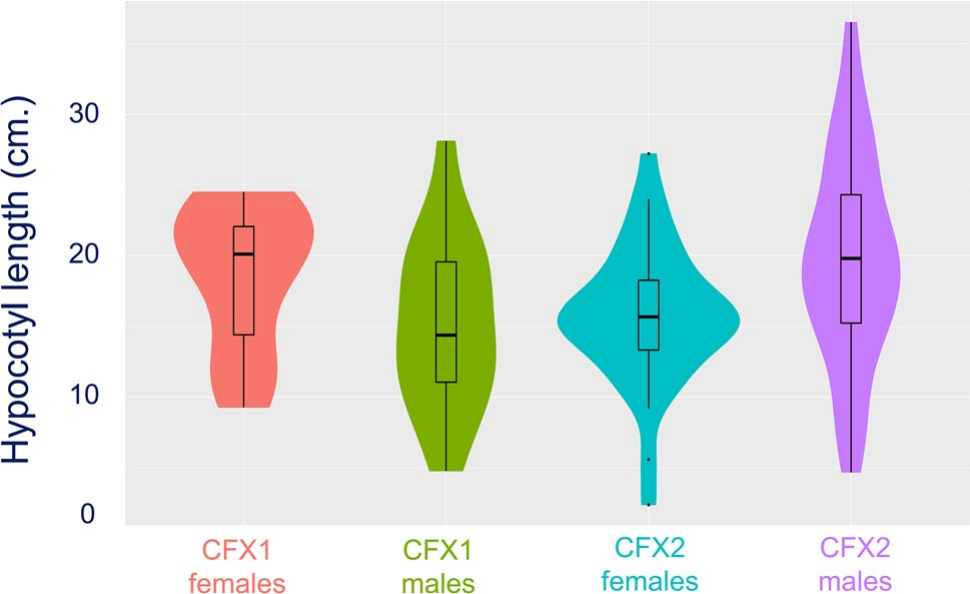
Morphological differences between two-week old male and female (hermaphrodite) plants for hypocotyl length. This violin plot represents the distribution of the phenotypes; their medians are indicated by the thick black line, their interquartile ranges are represented by the black rectangle and the distribution is shown by the coloured area.

Our models detected a significant interaction of sex by cultivar for two traits, collectively, in a MANOVA and, individually, in follow-up ANOVAs Type 3 SS (Table 1, Fig. 1). On average, male CFX-2 plants had longer hypocotyls than female CFX-2 plants (*post-hoc* comparison; P = 0.04; Δ = 0.62, 95% C.I. = [0.02,1.22]), but the same was not true for CFX-1 plants (P > 0.05). Further, there was a significant sex by cultivar interaction for height: but the *post-hoc* comparison was not significantly different. However, once Benjamini–Hochberg adjustments to p-values were applied, there were no significant differences in height among males and females within any cultivar. There were no significant differences or significant sex by cultivar differences in epicotyl length or petiole length, although epicotyls were longer in CFX-2, while petioles were longer in CFX-1.

**Table 1:** Multivariate analysis of variance (MANOVA) comparing the physical differences in three traits between male and female plants (factor: sex) of two cultivars (CFX-1, CFX-2) of hemp.

Model	Factor	df	F	P
(A) MANOVA	Sex	4	0.96	0.44
	Cultivar	4	8.25	< 0.001
	Sex × Cultivar	4	3.64	0.008
	Error	93		
(B) ANOVA—Height	Sex	1	1.37	0.25
	Cultivar	1	0.64	0.43
	Sex × Cultivar	1	4.00	0.05
	Error	97		
(C) ANOVA—Hypocotyl	Sex	1	2.02	0.16
	Cultivar	1	0.55	0.46
	Sex × Cultivar	1	7.25	0.008
	Error	96		
(D) ANOVA—Epicotyl	Sex	1	0.69	0.41
	Cultivar	1	5.62	0.02
	Sex × Cultivar	1	0.88	0.35
	Error	96		
(E) ANOVA—Petiole length	Sex	1	0.72	0.40
	Cultivar	1	16.30	< 0.001
	Sex × Cultivar	1	0.001	0.98
	Error	97		

Analysis of variance (ANOVA) was used to compare combinations of experimental factors (sex, cultivar and their combination). Response variables (four phenotypic traits: height, hypocotyl length, epicotyl length, petiole length) were natural log transformed. F-value is the test statistic used, p-value is the related probability of the null hypothesis being true based on the analysed observations, and df is the associated degrees of freedom.

### Distinguishing among co-sexual vs male or female *Cannabis* seedlings

**Figure 2:**
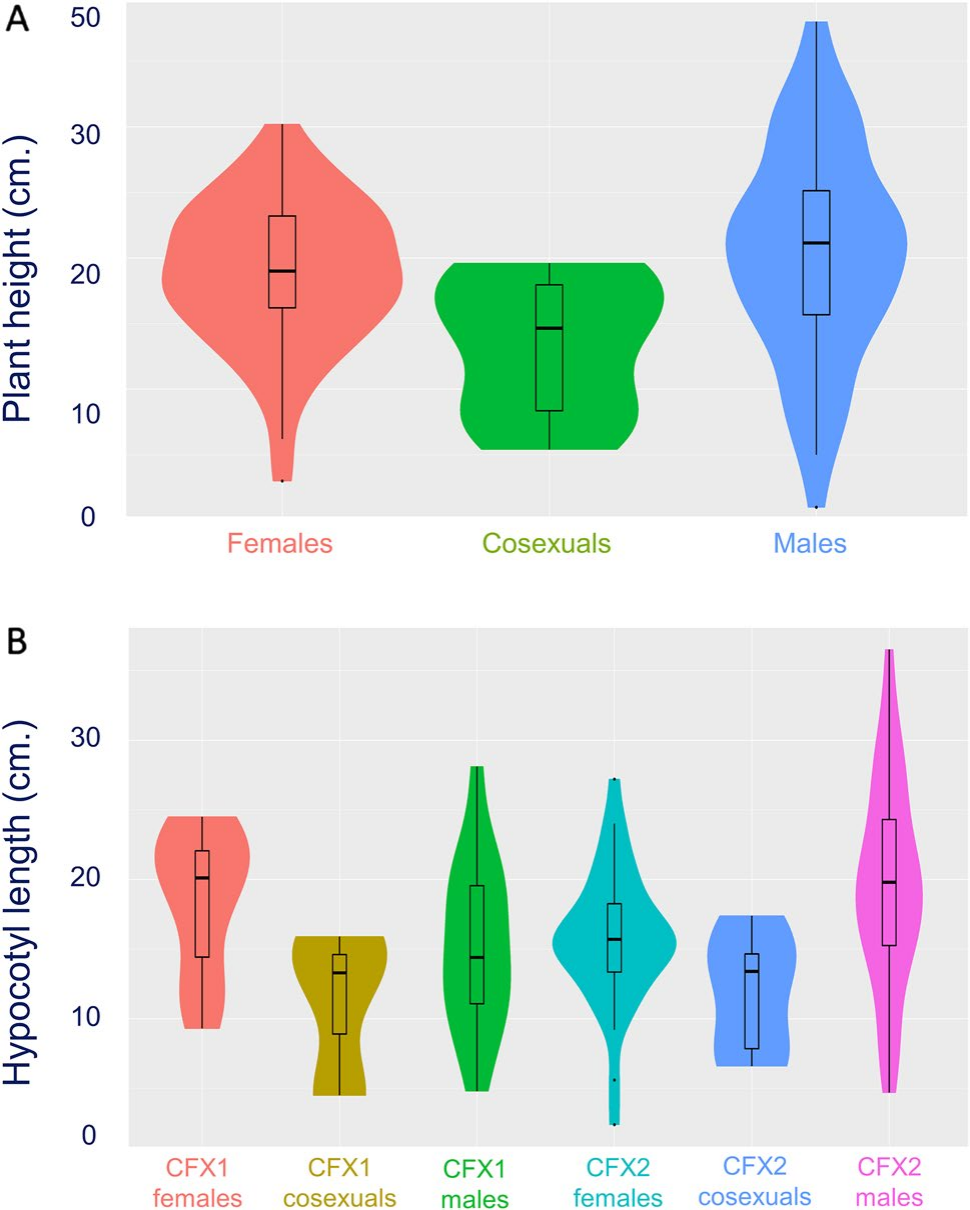
Morphological differences between two-week old male, female, and co-sexual (hermaphrodite) plants for (**a**) height and (**b**) hypocotyl length and (**c**) petiole length. This violin plot represents the distribution of the phenotypes; their medians are indicated by the white square, their medians are indicated by the thick black line, their interquartile ranges are represented by the black rectangle and the distribution is shown by the coloured area. Only traits that significantly affected sex or sex by cultivar interactions are shown here.

Only ten plants expressed co-sexuality in our study. We report these preliminary results with a caveat around the small sample size with the hope that this encourages more research on this topic. Co-sexual plants were significantly shorter in height than male and female plants (post-hoc comparisons to male (post-hoc comparison: P_height_ = 0.01; Δ = 0.99; 95% C.I. = [0.19, 1.78]; female: P_height_ = 0.06, Δ = 0.76; 95% C.I. = [0.03,1.56]; Benjamini–Hochberg adjustments to p-values for multiple hypothesis testing; Table 2, Fig. 2). The difference in height appears to be driven by co-sexuals expressing significantly shorter hypocotyls than males and females (*post-hoc* comparison to male: P_Hypocotyl_ = 0.005; Δ = 1.04, 95% C. I. = [0.27,1.82]; female: P_Hypocotyl_ = 0.05; Δ = 0.77; 95% C. I. = [0.01, 1.55]; Table 2, Fig. 2). There were no significant differences in height or hypocotyl length among cultivars detected in this analysis. There were no significant differences among the sexes or significant sex by cultivar interactions for epicotyl length, although there were significant differences in epicotyl length between the cultivars used, where CFX-2 grew epicotyls that were 39% longer than CFX-1. Finally, although there was a significant sex by cultivar interaction for petiole length, when we controlled for false discovery rates with Benjamini–Hochberg methods, we found no significant differences of interest (i.e., significant gender differences within cultivars). CFX-1 produced petioles that were 64% longer than CFX-2.

**Table 2:** Preliminary analysis comparing the physical differences in three traits between co-sexual, male and female plants in a multivariate analysis of variance (MANOVA) of two cultivars (CFX-1, CFX-2) of hemp.

Model	Factor	df	F	P
(A) ANOVA—Height	Sex	2	4.51	0.01
	Cultivar	1	0.69	0.41
	Sex × Cultivar	2	2.06	0.13
	Error	105		
(B) ANOVA—Hypocotyl	Sex	2	5.30	0.006
	Cultivar	1	0.57	0.45
	Sex × Cultivar	2	3.74	0.03
	Error	104		
(C) ANOVA—Epicotyl	Sex	2	2.07	0.13
	Cultivar	1	4.49	0.04
	Sex × Cultivar	2	0.59	0.55
	Error	104		
(D) ANOVA—Petiole length	Sex	2	0.94	0.39
	Cultivar	1	22.94	< 0.001
	Sex × Cultivar	2	3.90	0.02
	Error	105		

Analysis of variance (ANOVA) was used to compare combinations of experimental factors (sex, cultivar and their combination). Response variables (four phenotypic traits: height, hypocotyl length, epicotyl length, petiole length) were natural log transformed. F-value is the test statistic used, p-value is the related probability of the null hypothesis being true based on the analysed observations, and df is the associated degrees of freedom.

## Discussion

At a very early age of development, the average CFX-2 male *C. sativa* seedling grew 11% longer hypocotyls than female seedlings, potentially suggesting that sexual dimorphism in seedlings is visible early in the life cycle of this cultivar and may reflect important differences in the cost of reproduction later in life. Interestingly, CFX-1 plants did not show any statistically significant sexually dimorphic phenotypic variation between sexes and therefore the traits we measured do not vary consistently across cultivars among sexes. Given that *C. sativa* expresses adult sex-specific phenotypic differences, we predict the origin of the observed differences may be attributable to flowering phenology (timing of anthesis, in particular), rather than differential investment in vegetative growth. At flowering, female *C. sativa* plants are typically much more robust (wider stems, denser inflorescences) than male plants^53^ and we predict this morphological differentiation at maturity is a consequence of flowering time. These sex-specific differences in morphology in *C. sativa* may translate to differences in competitive ability, as described in seedlings of *Distichlis spicata*^44^ and *Silene alba*^54^. Male plants tend to flower earlier at a smaller biomass on average, than females. We will next explore the potential for gender-specific differences in seed morphology and germination, given that seed morphology can reflect the evolutionary history of *C. sativa* cultivars^55^ and sex-specific differences have been detected in the seeds and seedlings of other dioecious species^44,45^. Until we are able to further resolve sexual dimorphism in *C. sativa* seedlings, molecular markers may continue to serve as useful laboratory tools to discern the sex of male and female (but not co-sexual) seedlings when measuring the influence of sexual dimorphism in physiology, life history and biotic interactions^11^.

Our study provides only preliminary data for co-sexuals indeed. However, this small dataset provides, to our knowledge, the first insights on distinguishing co-sexual plants using seedling morphological features in the scholarly literature (although see^11^ for description of impact on floral morphology). Co-sexual plants cause significant economic losses in industrial facilities because they largely appear female but release small amounts of pollen, usually undetected and pollination lowers cannabinoid concentrations^56^. Pollination in Canadian facilities renders the floral material less valuable since only unpollinated floral material can be sold unmanipulated, and the plant material with lower cannabinoid concentrations must be sold as an oil-extracted product. Yet, to our knowledge, co-sexual plants have rarely been studied in modern scientific literature. The average co-sexual plant in our small sample was 49% shorter than the average unisexual plant. We predict the reduced size of co-sexual plants may reflect the reproductive cost of producing both pollen and ovules. To date, the only published methods for diagnosing sex in this dioecious species rely on floral morphology^57^, or height^58^, but the latter relies on subjective measures of relative height and calyx position, a floral feature. Because male flowers on co-sexual plants are often hidden within a dense female inflorescence (Wizenberg, Campbell pers. obs.), identification of co-sexual plants often occurs after anthesis and thus too late to prevent pollination in industrial contexts. It has been difficult to build knowledge on the morphological cues of sexually immature individuals; it is rare for this data to be collected and published, perhaps because early publications reported a lack of phenotypic differences among sexes in some dioecious plant species^47^. We encourage future research to compare early male and female traits in C. sativa and other species to improve our understanding of the ecology and evolution of dioecious plants^47,48,59,60^. This project provides critical information on the timing of onset of sex-specific morphological differences in non-reproductive traits of one dioecious plant species.

## Conclusions

Although there has been significant effort invested to develop methods that manipulate the expressed sex of *C. sativa*^61–67^, the natural process of sex determination in this plant is under-studied. Molecular markers have been developed as tools^68–70^, but the necessary machinery is generally inaccessible to farmers and as a consequence, farmers must rely on personal experience for distinguishing male, female, and co-sexual plants before flowering^58^. All existing citizen science developed tools require looking for under-developed flowers (Campbell pers. obs.). Since pollination has significant negative consequences for cannabinoid production^56^, a lack of knowledge of sex can be costly for the farmer in this expanding agricultural business, providing practical reasons for exploring this information.

## Methods

We collected data on phenotypic markers that can be used to predict the type (male, female or co-sexual) of flower produced by *C. sativa* plants during the vegetative stages of an experiment that measured the influence of UV-light on adult floral morphology and phytochemistry^71^. As such, the horticultural methods are described elsewhere but we summarize them here to show that the UV-lighting treatments were imposed well after the morphological measurements were collected. We also ran subsequent analyses to ensure that lighting did not cause enough stress to bias sex ratios once under those lighting treatments^71^.

### Plant cultivars and horticulture

This work was completed under the conditions of a federal hemp research license #18-C0169-R-01 and a federal marijuana research license #LIC-U5GX543XM6-2019. Two cultivars, CFX-1 and CFX-2 (Hemp Genetics International, Saskatoon, Saskatchewan, Canada) were included in both experiments, to provide the opportunity to measure phenotypic differences in sexual dimorphism. We planted hemp seeds without regard for their sexual expression in two batches, once on August 24, 2018, and again on September 3, 2018, in SC10 cone-tainers (Stuewe & Sons Inc., Tangent, Oregon, USA) with a density of one seed per pot. From the two batches, our dataset included 33 CFX-1 and 80 CFX-2 seedlings seven days after planting in Pro-mix BX HP mycorrhizae peat moss (Premier Tech Horticulture, Rivière-du-Loup, Quebec, Canada). These sample sizes were limited because of our research license, which limits the number of flowering adults at any time to 30 and number of vegetative plants to 150. Morphological data collection began at this point (week 1) and continued for two more weeks. All plants were given 250 mL of filtered water (Milli-Q water filtration system, Millipore Sigma, Burlington, Massachusetts, USA) three times per week, and fertilized weekly with 0.405% Miracle-Gro (10–10-10 NPK, Scotts, Marysville, Ohio, USA). Plants were grown in a common garden indoors, and spent the first week of growth under LED lights (65 W) and then switched to high pressure sodium lights (500 W) under 24-h light cycles. Oscillating fans were used to harden stem growth.

### Data collection

Between 5 and 7 days after germination, we began to measure a variety of morphological features of each plant. We measured the height of the first branch from the top of the soil and counted the number of fan leaves and branches. We measured the length of the epicotyl (first internode above the cotyledons), the length of the hypocotyl (the embryonic stem), and the length of the second internode. We also measured the length and width at the widest point of the longest fan leaf blade present on each plant, as well as the length of the petiole associated with that fan leaf blade. Two weeks after planting, we measured stem diameter at the base of the plant (VWR calipers #36934-154, accuracy: ± 0.2 mm, resolution: 0.1 mm). To measure height, we recorded the stem length from the base of the plant to the tallest meristem. Upon flowering (which started in week 3 for males), the sex of the plant was determined. We thus focus on phenotypic indicators prior to week 3 to minimize the likelihood of pollen release within a growth facility. After plants had begun flowering (4–6 weeks post germination), gender was diagnostically assigned using floral morphology.

### Statistical analysis

Of the 112 *C. sativa* plants grown in this study, 50 were female, 10 were co-sexual (where plants produced predominantly female flowers but at least one male flower) and 52 were male. Co-sexual plants were excluded from the first stage of analysis due to small sample size. To determine which (if any) morphological features differed between male and female plants during vegetative growth, we first analyzed trait values obtained 2 weeks after germination (September 12th, 2018) (See Supplementary Information Part 1 for statistical code). We characterized response variable distributions using the hist() function (R stats package; R core team, 2019) to determine if traits were sufficiently parametric. Any traits that were insufficiently parametric underwent logarithmic transformation to adhere to assumptions of normality. Following this, we used multivariate analysis of variance, the manova() function (R stats package v. 4.0.2 (2020-06-22); R core team, 2019), to determine if any phenotypic traits differed among subgroups, using genotype, sex, and their interaction as main effects. Next, we used univariate analysis of variance, the aov() function (R stats package; R core team, 2019), to determine which phenotypic traits showed significant main effects for sex, or the genotype by sex interaction. Any traits identified as being marginally or statistically significantly different between subgroups underwent subsequent *post-hoc* analysis, using the Tukey’s Honest Significant Differences test, the TukeyHSD() function (R stats package; R core team, 2019), and pairwise t-tests with a Benjamini–Hochberg adjustment, the pairwise.t.test() function (R stats package; R core team, 2019).

To determine if any morphological traits could distinguish between unisexuals (male or female) and co-sexuals, we performed a second stage of analysis which included the 10 co-sexual plants as a third functional gender. Due to the small sample size (10 hermaphroditic plants), we were unable to perform multivariate analysis of variance due to insufficient degrees of freedom and instead used only univariate analysis of variance (See Supplementary Information Part 2 for statistical code). Analysis followed the same procedure as above, using the aov() function, TukeyHSD() and pairwise.t.test() for post hoc analysis with Benjamini–Hochberg adjustments for multiple hypothesis testing^72^.

## Supplementary Information

Supplementary Information 1.

Supplementary Information 2.
