# Supplementary material for: Dioecious hemp (Cannabis sativa L.) plants do not express significant sexually dimorphic morphology in the seedling stage
Source: Sci Rep. 2021 Aug 19;11:16825. doi: 10.1038/s41598-021-96311-w (PMC8376874; doi:10.1038/s41598-021-96311-w)
Supplement: Supplementary file 2 — Supplementary Information 2. [file 41598_2021_96311_MOESM2_ESM.docx]

*Supplemental Materials for:*

**Dioecious hemp (*Cannabis sativa* L.) plants do not express statistically significant sexually dimorphic morphology in the seedling stage.**

**Lesley G. Campbell^1^, Kristen Peach^2^, and Sydney B. Wizenberg^1^**

**Part 1**. R Code to analyse phenotypic comparisons between male and female plants.

data = na.pass(read.csv(file="C:/Users/sydne/Desktop/SexData.csv", TRUE, ","))

# factors

genotype = as.factor(data$Genotype)

sex = as.factor(data$Sex)

geno_sex = as.factor(data$Geno_Sex)

# standardizing response variables, log transforming epicotyl

height = scale(data$X2_Height)

hypocotyl = scale(data$X2_Hypocotyl)

epicotyl = scale(log(data$X2_Epicotyl))

petiole = scale(data$X2_Petiole)

#manova

manova1 = manova(cbind(height, hypocotyl, epicotyl, petiole) ~ sex*genotype)

summary(manova1)

# height, anova

anova1 = aov(height ~ sex*genotype)

summary(anova1)

# height, post hoc analysis

TukeyHSD(aov(height ~ sex*genotype))

pairwise.t.test(height, geno_sex, p.adjust.method = "BH")

# hypocotyl, anova

anova2 = aov(hypocotyl ~ sex*genotype)

summary(anova2)

# hypocotyl, post hoc analysis

TukeyHSD(aov(hypocotyl ~ sex*genotype))

pairwise.t.test(hypocotyl, geno_sex, p.adjust.method = "BH")

# epicotyl, anova

anova3 = aov(epicotyl ~ sex*genotype)

summary(anova3)

# petiole, anova

anova4 = aov(petiole ~ sex*genotype)

summary(anova4)

**Part 2**. R Code to analyse phenotypic comparisons between male, female and cosexual plants.

data = na.pass(read.csv(file="C:/Users/sydne/Desktop/SexData_H.csv", TRUE, ","))

# factors

genotype = as.factor(data$Genotype)

sex = as.factor(data$Sex)

geno_sex = as.factor(data$Geno_Sex)

# standardizing response variables, log transforming petiole

height = scale(data$X2_Height)

hypocotyl = scale(data$X2_Hypocotyl)

epicotyl = scale(data$X2_Epicotyl)

petiole = scale(log(data$X2_Petiole))

# manova, doesnt work because of insufficient degrees of freedom for the

# hermaphrodite subgroups

# manova1 = manova(cbind(height, hypocotyl, epicotyl) ~ sex*genotype)

# summary(manova1)

# height, anova

anova1 = aov(height ~ sex*genotype)

summary(anova1)

# height, post hoc analysis

TukeyHSD(aov(height ~ sex))

pairwise.t.test(height, sex, p.adjust.method = "BH")

# hypocotyl, anova

anova2 = aov(hypocotyl ~ sex*genotype)

summary(anova2)

# hypocotyl, post hoc analysis

TukeyHSD(aov(hypocotyl ~ sex*genotype))

pairwise.t.test(hypocotyl, sex, p.adjust.method = "BH")

pairwise.t.test(hypocotyl, geno_sex, p.adjust.method = "BH")

# epicotyl, anova

anova3 = aov(epicotyl ~ sex*genotype)

summary(anova3)

# petiole, anova

# R doesnt like the pre-standardized petiole data for ???unknown?? reason

# so i reimported it directly as the response for this model

anova4 = aov(scale(data$X2_Petiole) ~ sex*genotype)

summary(anova4)

#petiole, post hoc analysis

TukeyHSD(aov(scale(data$X2_Petiole) ~ sex*genotype))

pairwise.t.test(height, geno_sex, p.adjust.method = "BH")
